# Influence of the nickel-titanium alloy components on biological functions

**DOI:** 10.1186/1753-6561-5-S1-P79

**Published:** 2011-11-22

**Authors:** Akiko Ogawa, Ryota Akatsuka, Hidekazu Tamauchi, Katsuya Hio, Hideyuki Kanematsu

**Affiliations:** 1 Suzuka National College of Technology, Suzuka, Japan, 510-0294; 2 Mie Prefecture Industrial Research Institute Metal Science Branch, Kuwana, Japan, 511-0937; 3 Kitasato University School of Medicine, Sagamihara, Japan, 225-8555

## Background

Nickel-titanium alloy is applied to biomaterials such dental wire, stents and so on, because they have outstanding capacities for corrosion-resistant, adequate mechanical strength and shape-memory [1,2]. On the other hand they have a risk of metal allergy because of leaking nickel ions from them in aqueous conditions. It is not clear the reason why nickel causes allergy and what the rate of components is suitable for biomaterial therefore we have to design biocompatible nickel-titanium alloys based on experiences. In order to create biological friendly nickel-titanium alloys more effectively, it is necessary to make the relationship clear between characters of nickel-titanium alloys and biological reactions. In this study, we investigated 1) the material properties of nickel-titanium alloys, 2) the effect of these alloys on cellular function and 3) the allergy test for these alloys.

## Materials and methods

Titanium particles were mixed with nickel particles to become 5%, 15%, 25% or 50% titanium by weight and the nickel-titanium alloys were made by arc melting. The components of nickel-titanium alloy were determined by X-ray fluorescence analysis. Nickel plate was used as a control. Nickel plate and nickel-titanium alloys were cut into determined surface area by sharing machine then soaked in 70% ethanol due to sterilize. The sterilized ones were utilized for cellular assay. First, they were soaked in PBS and heated at 36.5 °C for 25 hours in order to make extracts. Next, each extract was added to the culture supernatant of MOLT-3 cells (Riken bioresource center cell bank, Japan) and the MOLT-3 cells were cultured with 10% FBS containing RPMI for 5 days at 36.5 °C in humidified air containing 5% of CO_2_. After that, the viable cell numbers were determined by the trypan blue exclusion assay and counting in a hemacytometer under a phase contrast microscope. Both nickel and titanium concentrations of the extracts were determined by ICP atomic emission spectrometer (Shimadzu, Japan). Nickel-titanium alloys were shaped into round columns (φ: 10 mm, depth: 5 mm) then sterilized and transplanted to dorsal subcutaneously of mice. After 28 days, a certain amount of nickel solution was injected into the auricularis skin of the mice and the degree of turgid auriculae was measured.

## Results

**Figure 1:**
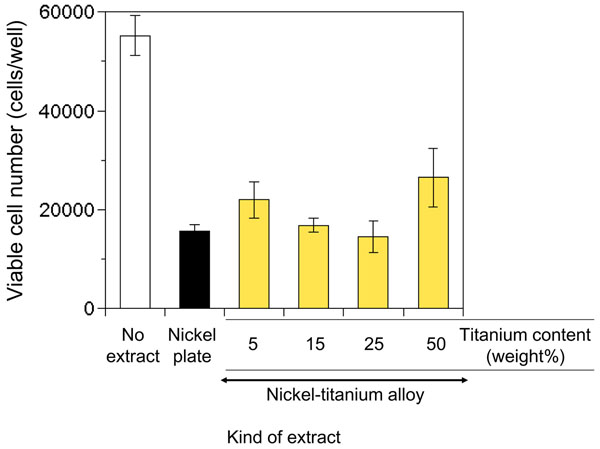
Effect of extract of nickel-titanium alloys on cell survival of MOLT-3 cells. MOLT-3 cells were seeded in 12-well plate at 96,800 cells/well (n =4). Next day, each extract of nickel-titanium alloy or nickel plate was added to the culture supernatant.

In cellular assay with MOLT-3, the viable cell number of the cells cultured with the extract of nickel-5% or 50% titanium alloy was significantly more than that of nickel plate (Figure 1). In allergy test, transplanting nickel-50% titanium alloy indicated the lower degree of turgid auriculae than transplanting nickel plate in a mouse but nickel-5% or 15% titanium alloy did not. About nickel concentration of the extract, nickel-50% titanium alloy was the lowest and nickel-25% titanium alloy was the highest.

## Conclusions

We studied the relationship between characters of nickel-titanium alloys and biological reactions. Four kinds of nickel-titanium alloys having different titanium contents were tested. Except nickel-50% titanium alloy, the nickel concentration of the extract of nickel-titanium alloy increased with the content rate of titanium. This result suggests that increasing the content rate of titanium will promote leaking nickel from nickel-titanium alloys. Otherwise the viable cell number decreased with the content rate of titanium. The extract of nickel-50% titanium alloy indicated not only the largest viable cell number among all extracts but also the lowest nickel concentration. These results indicate that the amount of nickel would affect cell survival of MOLT-3 cells, and that nickel concentration of the extracts was not correlated with the titanium content rates of nickel-titanium alloys but it was done with the structural states of them. When nickel-titanium alloys were transplanted into mice, only nickel-50% titanium alloy affected the allergic reaction for the better. This result suggests that cellular assay will be more sensitive to detect the material differences of nickel-titanium alloys than animal test.
